# Supplementary figures and images for: EEG and ERP biosignatures of mild cognitive impairment for longitudinal monitoring of early cognitive decline in Alzheimer’s disease
Source: PLoS One. 2024 Aug 8;19(8):e0308137. doi: 10.1371/journal.pone.0308137 (PMC11309464; doi:10.1371/journal.pone.0308137)

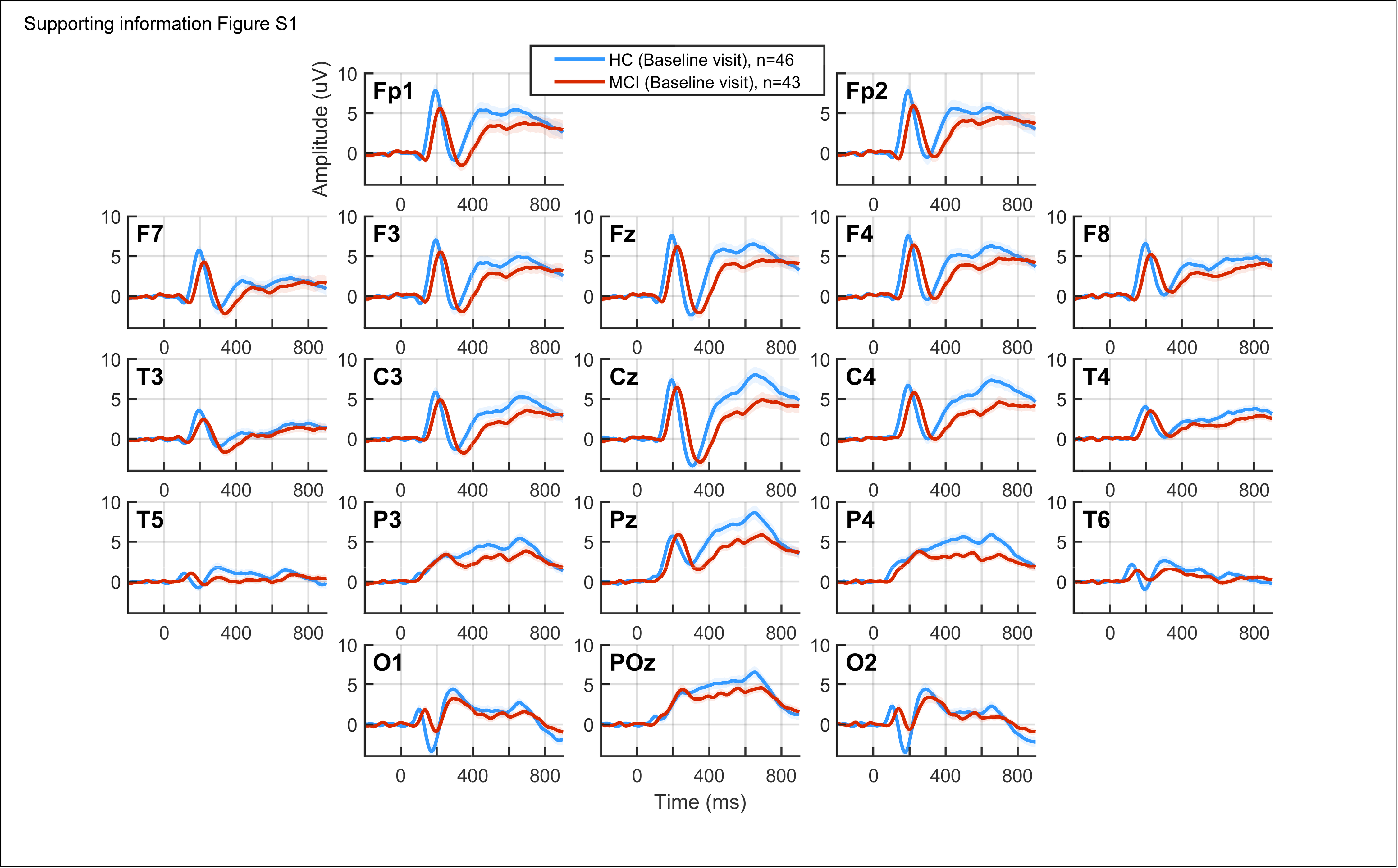

Supplement: S1 Fig — (TIF) [file pone.0308137.s001.tif]

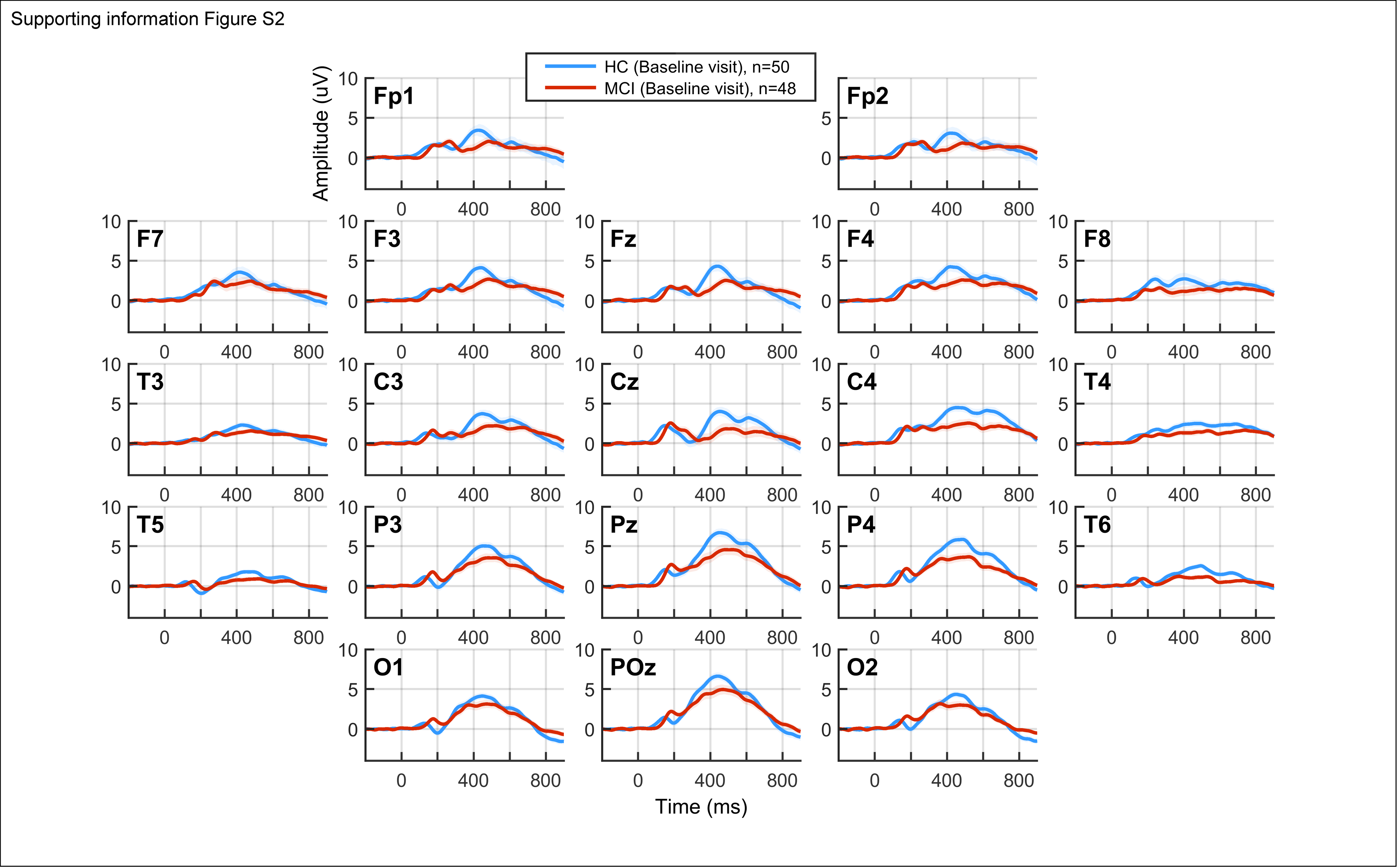

Supplement: S2 Fig — (TIF) [file pone.0308137.s002.tif]
