# Supplementary material for: EEG and ERP biosignatures of mild cognitive impairment for longitudinal monitoring of early cognitive decline in Alzheimer’s disease
Source: PLoS One. 2024 Aug 8;19(8):e0308137. doi: 10.1371/journal.pone.0308137 (PMC11309464; doi:10.1371/journal.pone.0308137)
Supplement: S1 Table — (DOCX) [file pone.0308137.s003.docx]

| **Classification Model** | **SVM ^[1]^** | **LDA ^[2]^** | **Logit ^[3]^** | **KNN ^[4]^** | **Tree ^[5]^** |
| --- | --- | --- | --- | --- | --- |
| Accuracy | 78% | 80% | 77% | 77% | 66% |
| AUC | 0.88 | 0.88 | 0.81 | 0.83 | 0.7 |
| Legend:  All performance reported are performance metrics in 5-fold cross validation.  **^[1]^** SVM (Support Vector Machine; Linear Kernel function)  **^[2]^** LDA (Linear Discriminant Function, Support Vector Machine; Linear Kernel function)  **^[3]^** Logit (Logistic Regression)  **^[4]^** KNN (K Nearest Neighbors; K=5)  **^[3]^** Classification Decision Tree (max number of splits = 20) | | | | | |
